# Supplementary material for: Regulation of two motor patterns enables the gradual adjustment of locomotion strategy in Caenorhabditis elegans
Source: eLife. 2016 May 25;5:e14116. doi: 10.7554/eLife.14116 (PMC4880447; doi:10.7554/eLife.14116)
Supplement: Source code 1. — See ‘readme.txt’ for an overview. DOI: http://dx.doi.org/10.7554/eLife.14116.023 [file elife-14116-code1.zip › HumsSourceCode/Eigenmovie/VideoUtils_v1_2_4/html/example_MultibandBlending.html]

MultibandBlending example 

# MultibandBlending example

Example of how to use the class to perform a Multi-Band Blending. It is based on the paper: P. BURT and E. ADELSON, A Multiresolution Spline with Application to Image Mosaics, Acm Transactions on Graphics, vol. 2, no. 4, pp. 217-236, 1983.

## Contents

- Open load the images
- Initialize the MultibandBlending object
- Strich the images
- Show results

## Open load the images

First of all we have to load the mask and the images that we want to fuse.

```
I1    = double(imread('./Resources/TestFace_1.jpg')) / 255;
I2    = double(imread('./Resources/TestFace_2.jpg')) / 255;
Imask = double(imread('./Resources/TestMask.png'  )) / 255;

mask = Imask(:, :, 1);
```

## Initialize the MultibandBlending object

In order to initialize the object we can define the number of pyramid levels and the blur efect of the mask.

```
mb = MultibandBlending('NumLevels', 4, 'MaskBlur', 26);
```

## Strich the images

We sttich the images using the stitchMask function.

```
res1 = mb.stitchMask(I1, I2, mask);
res2 = mb.stitchMask(I2, I1, mask);
```

## Show results

Finally we show the results.

```
sizeIm  = size(I1);
newSize = sizeIm;
newSize(2) = newSize(2) * 2;
newSize(1) = newSize(1) * 2;

finalIma = zeros(newSize);

finalIma(       1:sizeIm(1),       1:sizeIm(2), :) = I1;
finalIma(       1:sizeIm(1), sizeIm(2) + 1:end, :) = I2;
finalIma( sizeIm(1) + 1:end,       1:sizeIm(2), :) = res2;
finalIma( sizeIm(1) + 1:end, sizeIm(2) + 1:end, :) = res1;

finalIma(:, sizeIm(2) - 1:sizeIm(2) + 2, 1) = 1;
finalIma(:, sizeIm(2) - 1:sizeIm(2) + 2, 2) = 0;
finalIma(:, sizeIm(2) - 1:sizeIm(2) + 2, 3) = 0;

finalIma(sizeIm(1) - 1:sizeIm(1) + 2, :, 1) = 1;
finalIma(sizeIm(1) - 1:sizeIm(1) + 2, :, 2) = 0;
finalIma(sizeIm(1) - 1:sizeIm(1) + 2, :, 3) = 0;

figure, imshow(finalIma);
```

```
Warning: Image is too big to fit on screen; displaying at 50%
```

Published with MATLAB® 7.13
