# Supplementary material for: Regulation of two motor patterns enables the gradual adjustment of locomotion strategy in Caenorhabditis elegans
Source: eLife. 2016 May 25;5:e14116. doi: 10.7554/eLife.14116 (PMC4880447; doi:10.7554/eLife.14116)
Supplement: Source code 1. — See ‘readme.txt’ for an overview. DOI: http://dx.doi.org/10.7554/eLife.14116.023 [file elife-14116-code1.zip › HumsSourceCode/Eigenmovie/VideoUtils_v1_2_4/html/example_VideoLightCorrector.html]

VideoLigthCorrector example 

# VideoLigthCorrector example

It is usual to have fast light changes in some video, which can cause problems in computer vision algorithms. In here we show an example which prevent this light changes by reducing the intensity differences between consecutive frames using a polynomial approach.

## Contents

- Create the VideoLightCorrector object
- Reproduce the Video Sequence
- Release the VideoLightCorrector

## Create the VideoLightCorrector object

In order to create the object we have to define the number of degrees of the polynomial function.

```
degree = 6; % polynomial degree

vp = VideoLightCorrector('./Resources/TestVideo.mp4', degree);
```

## Reproduce the Video Sequence

After we have processed the video we are ready to reproduce it in the same way as a VideoPlayer object.

```
while (true)
    plot(vp);

    drawnow;

    if ( ~vp.nextFrame )

        break;
    end
end
```

## Release the VideoLightCorrector

Finally it is necessary to release the object.

```
clear vp;
```

Published with MATLAB® 7.13
