# Supplementary material for: Regulation of two motor patterns enables the gradual adjustment of locomotion strategy in Caenorhabditis elegans
Source: eLife. 2016 May 25;5:e14116. doi: 10.7554/eLife.14116 (PMC4880447; doi:10.7554/eLife.14116)
Supplement: Source code 1. — See ‘readme.txt’ for an overview. DOI: http://dx.doi.org/10.7554/eLife.14116.023 [file elife-14116-code1.zip › HumsSourceCode/Eigenmovie/VideoUtils_v1_2_4/html/example_VideoPlayer.html]

Simple VideoPlayer Example 

# Simple VideoPlayer Example

In this example we show how to use the **VideoPlayer** object.

## Contents

- Create a new VideoPlayer Object
- Reproducing the video sequence
- Releaseing the VideoPlayer Object

## Create a new VideoPlayer Object

To generate a new **VideoPlayer** object we have to use the next sentence. Note that it is not necessary to include the parameters Verbose and Showtime, these parameters are obtional.

```
vp = VideoPlayer('./Resources/TestVideo.mp4', 'Verbose', false, 'ShowTime', false);
```

## Reproducing the video sequence

Then we have to create a loop to play the entire video sequence:

```
while ( true )
   plot( vp );


   % Your code here.
   % To access to the current frame use -> vp.Frame


   drawnow;
   if ( ~vp.nextFrame )
       break;
   end
end
```

## Releaseing the VideoPlayer Object

After we have used the **VideoPlayer** object it is necessary to release it using this command:

```
clear vp;
```

Published with MATLAB® 7.13
