# Supplementary material for: Regulation of two motor patterns enables the gradual adjustment of locomotion strategy in Caenorhabditis elegans
Source: eLife. 2016 May 25;5:e14116. doi: 10.7554/eLife.14116 (PMC4880447; doi:10.7554/eLife.14116)
Supplement: Source code 1. — See ‘readme.txt’ for an overview. DOI: http://dx.doi.org/10.7554/eLife.14116.023 [file elife-14116-code1.zip › HumsSourceCode/Eigenmovie/VideoUtils_v1_2_4/html/example_VideoPlayer_static_picture.html]

VideoPlayer Example using an static picture 

# VideoPlayer Example using an static picture

The **VideoPlayer** object is capable of creating an online video using an static picture

## Contents

- Create the VideoPlayer object
- Reproduce the synthetic video
- Release the VideoPlayer object

## Create the VideoPlayer object

In order to create a **VideoPlayer** object using an static picture we have to define the **VideoPlayer** object using the optional parameter 'UseStaticPicture', where we have define a projective transform, which will be applied to the image for each frame. See next source code:

```
pictureName = 'peppers.png'; % Static picture

transX = 10; % X Translation
transY = 5;  % Y Translation

rotX = 0;    % Rotation in the X axis (in degrees)
rotY = 0;    % Rotation in the Y axis (in degrees)
rotZ = 10;   % Rotation in the Z axis (in degrees)

scale = 1.08; % Scale factor

imageSize = [320, 240]; % Cutted region of the static picture

numberFrames = 10;      % Number of frames of the synthetic video.

vp = VideoPlayer(pictureName, ...
    'UseStaticPicture', [transX transY rotX rotY rotZ scale], ...
    'ValidRectangle', imageSize, 'MaxFrames', numberFrames);
```

## Reproduce the synthetic video

In order to reproduce the synthetic video you have to create the loop like in the example\_VideoPlayer.m.

```
while(true)
    plot(vp);

    disp( mat2str(vp.Tgp) );

    drawnow;

    if (~vp.nextFrame)
        break;
    end
end
```

```
[1 0 0;0 1 0;0 0 1]
[1.06359237325318 0.187540031880285 0;-0.187540031880285 1.06359237325318 0;10 5 1]
[1.09605747288468 0.39893229517506 0;-0.39893229517506 1.09605747288468 0;20 10 1]
[1.0909425934521 0.629856 0;-0.629856 1.0909425934521 0;30 15 1]
[1.04219500773272 0.874505446603326 0;-0.874505446603326 1.04219500773272 0;40 20 1]
[0.944465882331592 1.12557060835134 0;-1.12557060835134 0.944465882331592 0;50 25 1]
[0.793437161472 1.37427347628274 0;-1.37427347628274 0.793437161472 0;60 30 1]
[0.586162422042981 1.61046801869592 0;-1.61046801869592 0.586162422042981 0;70 35 1]
[0.321410658004117 1.82281042137011 0;-1.82281042137011 0.321410658004117 0;80 40 1]
[1.2240373090321e-16 1.99900462710443 0;-1.99900462710443 1.2240373090321e-16 0;90 45 1]
```

## Release the VideoPlayer object

Finally you have to release the object.

```
clear vp;
```

Published with MATLAB® 7.13
