# Supplementary material for: Regulation of two motor patterns enables the gradual adjustment of locomotion strategy in Caenorhabditis elegans
Source: eLife. 2016 May 25;5:e14116. doi: 10.7554/eLife.14116 (PMC4880447; doi:10.7554/eLife.14116)
Supplement: Source code 1. — See ‘readme.txt’ for an overview. DOI: http://dx.doi.org/10.7554/eLife.14116.023 [file elife-14116-code1.zip › HumsSourceCode/Eigenmovie/VideoUtils_v1_2_4/html/example_VideoPlayerGPyramid.html]

Simple VideoPlayerGPyramid Example 

# Simple VideoPlayerGPyramid Example

In here you can see and example of how to use the **VideoPlayerGPyramid** object in order to reproduce a Video as a gaussian pyaramid.

## Contents

- Create a new VideoPlayerGPyramid Object
- Define the position of the windows
- Play the video sequence
- Release the VideoPlayerGPyarmid Object

## Create a new VideoPlayerGPyramid Object

To generate a new **VideoPlayerGPyramid** object we have to use the next sentence, where 'levels' is the number of gaussian pyramid levels.

```
levels = 4; % Number of gaussian pyramid levels

vpgp = VideoPlayerGPyramid('./Resources/TestVideo.mp4', levels);
```

## Define the position of the windows

This part is optional but if you want to order the windows (one for each level of the gaussian pyramid) the you must do as follows:

```
startX = 100;
startY = 600;
width  = 466;
height = 350;

vpgp.setPosition ( startX, startY, width, height);
```

   

## Play the video sequence

Then you need to include this loop in order to play the entire video sequence:

```
while ( true )
   plot( vpgp );

   drawnow;
   if ( ~vpgp.nextFrame )
       break;
   end
end
```

   

## Release the VideoPlayerGPyarmid Object

Finally you have to release the object.

```
clear vpgp;
```

Published with MATLAB® 7.13
