# Supplementary figures and images for: Regulation of two motor patterns enables the gradual adjustment of locomotion strategy in Caenorhabditis elegans
Source: eLife. 2016 May 25;5:e14116. doi: 10.7554/eLife.14116 (PMC4880447; doi:10.7554/eLife.14116)

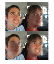

Supplement: Source code 1. — See ‘readme.txt’ for an overview. DOI: http://dx.doi.org/10.7554/eLife.14116.023 [file elife-14116-code1.zip › HumsSourceCode/Eigenmovie/VideoUtils_v1_2_4/html/example_MultibandBlending.png]

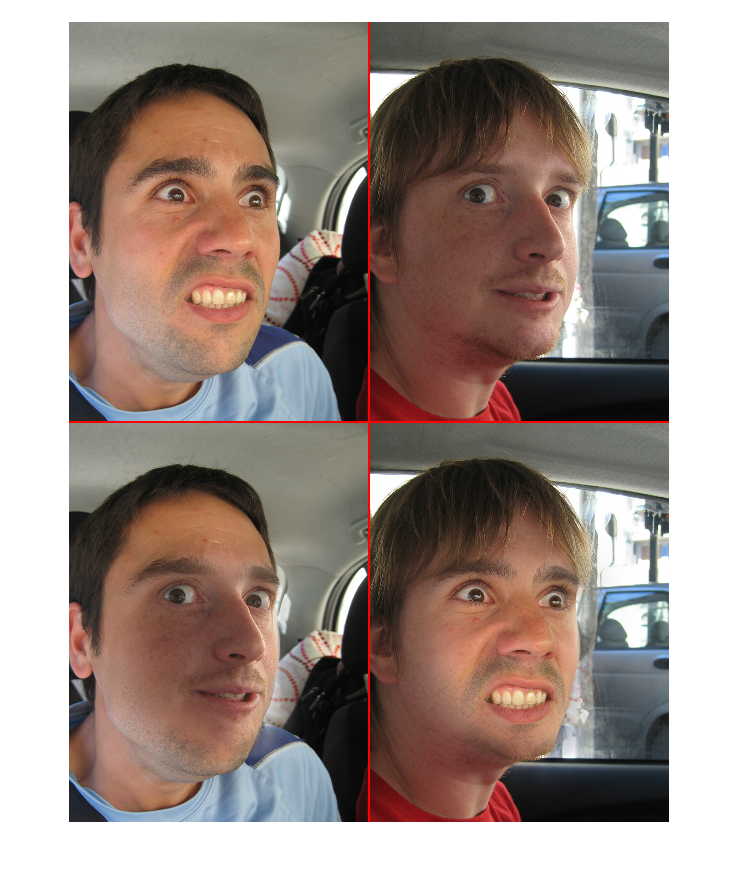

Supplement: Source code 1. — See ‘readme.txt’ for an overview. DOI: http://dx.doi.org/10.7554/eLife.14116.023 [file elife-14116-code1.zip › HumsSourceCode/Eigenmovie/VideoUtils_v1_2_4/html/example_MultibandBlending_01.png]

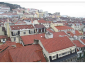

Supplement: Source code 1. — See ‘readme.txt’ for an overview. DOI: http://dx.doi.org/10.7554/eLife.14116.023 [file elife-14116-code1.zip › HumsSourceCode/Eigenmovie/VideoUtils_v1_2_4/html/example_VideoLightCorrector.png]

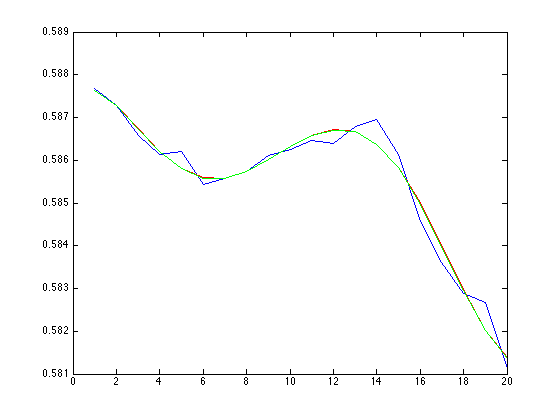

Supplement: Source code 1. — See ‘readme.txt’ for an overview. DOI: http://dx.doi.org/10.7554/eLife.14116.023 [file elife-14116-code1.zip › HumsSourceCode/Eigenmovie/VideoUtils_v1_2_4/html/example_VideoLightCorrector_01.png]

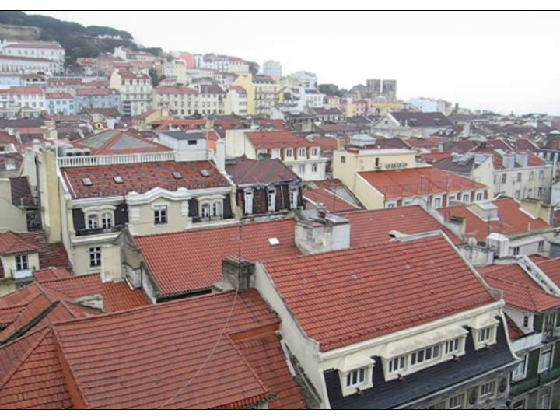

Supplement: Source code 1. — See ‘readme.txt’ for an overview. DOI: http://dx.doi.org/10.7554/eLife.14116.023 [file elife-14116-code1.zip › HumsSourceCode/Eigenmovie/VideoUtils_v1_2_4/html/example_VideoLightCorrector_02.png]

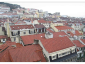

Supplement: Source code 1. — See ‘readme.txt’ for an overview. DOI: http://dx.doi.org/10.7554/eLife.14116.023 [file elife-14116-code1.zip › HumsSourceCode/Eigenmovie/VideoUtils_v1_2_4/html/example_VideoPlayer.png]

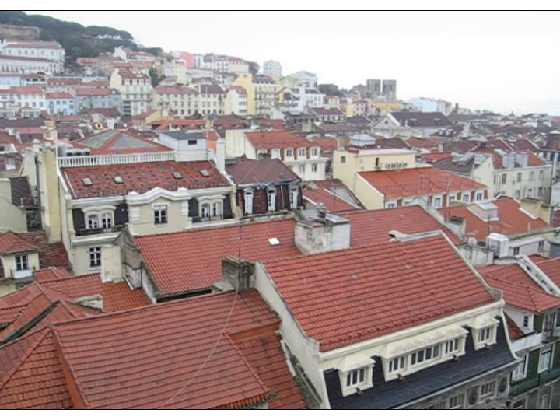

Supplement: Source code 1. — See ‘readme.txt’ for an overview. DOI: http://dx.doi.org/10.7554/eLife.14116.023 [file elife-14116-code1.zip › HumsSourceCode/Eigenmovie/VideoUtils_v1_2_4/html/example_VideoPlayer_01.png]

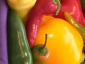

Supplement: Source code 1. — See ‘readme.txt’ for an overview. DOI: http://dx.doi.org/10.7554/eLife.14116.023 [file elife-14116-code1.zip › HumsSourceCode/Eigenmovie/VideoUtils_v1_2_4/html/example_VideoPlayer_static_picture.png]

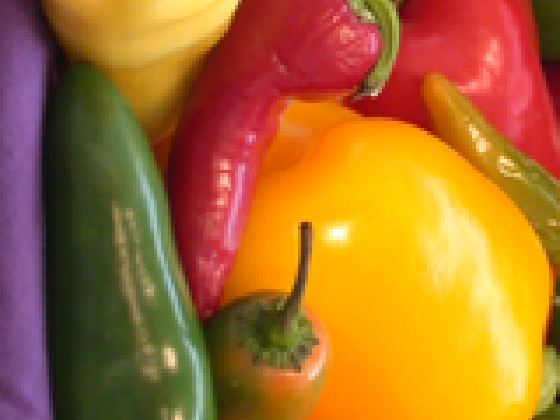

Supplement: Source code 1. — See ‘readme.txt’ for an overview. DOI: http://dx.doi.org/10.7554/eLife.14116.023 [file elife-14116-code1.zip › HumsSourceCode/Eigenmovie/VideoUtils_v1_2_4/html/example_VideoPlayer_static_picture_01.png]

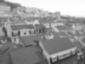

Supplement: Source code 1. — See ‘readme.txt’ for an overview. DOI: http://dx.doi.org/10.7554/eLife.14116.023 [file elife-14116-code1.zip › HumsSourceCode/Eigenmovie/VideoUtils_v1_2_4/html/example_VideoPlayerGPyramid.png]

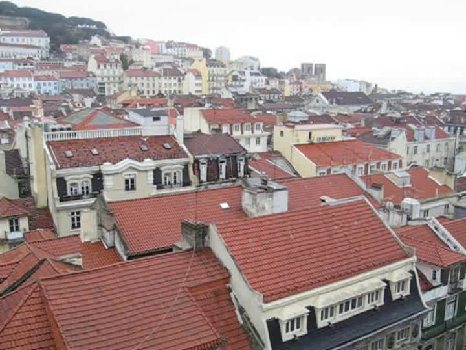

Supplement: Source code 1. — See ‘readme.txt’ for an overview. DOI: http://dx.doi.org/10.7554/eLife.14116.023 [file elife-14116-code1.zip › HumsSourceCode/Eigenmovie/VideoUtils_v1_2_4/html/example_VideoPlayerGPyramid_01.png]

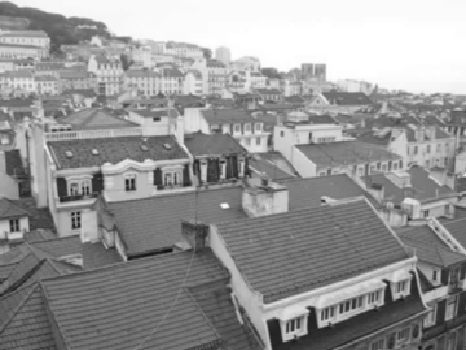

Supplement: Source code 1. — See ‘readme.txt’ for an overview. DOI: http://dx.doi.org/10.7554/eLife.14116.023 [file elife-14116-code1.zip › HumsSourceCode/Eigenmovie/VideoUtils_v1_2_4/html/example_VideoPlayerGPyramid_02.png]

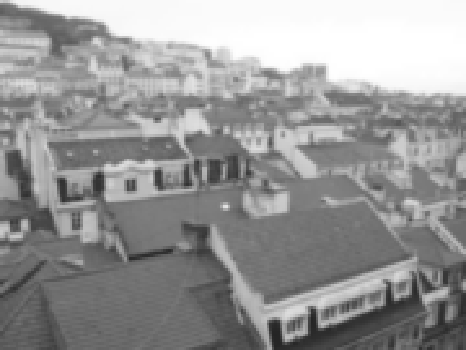

Supplement: Source code 1. — See ‘readme.txt’ for an overview. DOI: http://dx.doi.org/10.7554/eLife.14116.023 [file elife-14116-code1.zip › HumsSourceCode/Eigenmovie/VideoUtils_v1_2_4/html/example_VideoPlayerGPyramid_03.png]

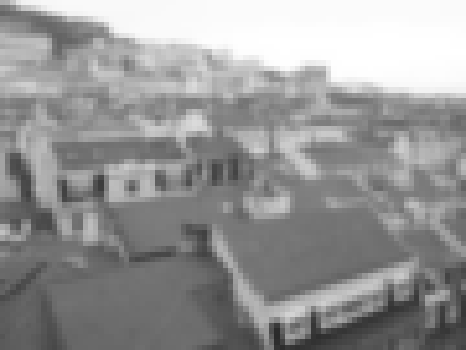

Supplement: Source code 1. — See ‘readme.txt’ for an overview. DOI: http://dx.doi.org/10.7554/eLife.14116.023 [file elife-14116-code1.zip › HumsSourceCode/Eigenmovie/VideoUtils_v1_2_4/html/example_VideoPlayerGPyramid_04.png]
