# Supplementary material for: Regulation of two motor patterns enables the gradual adjustment of locomotion strategy in Caenorhabditis elegans
Source: eLife. 2016 May 25;5:e14116. doi: 10.7554/eLife.14116 (PMC4880447; doi:10.7554/eLife.14116)
Supplement: Source code 1. — See ‘readme.txt’ for an overview. DOI: http://dx.doi.org/10.7554/eLife.14116.023 [file elife-14116-code1.zip › HumsSourceCode/Eigenmovie/VideoUtils_v1_2_4/html/example_VideoRecorder.html]

Simple VideoRecorder Example 

# Simple VideoRecorder Example

In here you can see and example of how to use the **VideoRecorder** object.

## Contents

- Create a new VideoRecorder Object
- Insert frames to the VideoRecorder Object
- Close the VideoRecorder Object
- See the Recorded Video

## Create a new VideoRecorder Object

First of all we have to define the video name and the format of this new video, in this example the video name is set to **NewVideo** and the format to **mov**.

```
vr = VideoRecorder('NewVideo', 'Format', 'mov');
```

## Insert frames to the VideoRecorder Object

After we have created the **VideoRecorder** object, we are ready to insert frames to this object.

```
vp = VideoPlayer('./Resources/TestVideo.mp4');

for i = 1:10
    vr.addFrame(vp.Frame);

    vp + 1;
end

clear vp;
```

## Close the VideoRecorder Object

Finally we have to close the video sequence by releasing the **VideoRecorder** object.

```
clear vr;
```

## See the Recorded Video

Finally with this script you can see the recorded video.

```
vp = VideoPlayer('NewVideo.mov');

while (true)
    plot(vp);
    drawnow;

    if (~vp.nextFrame)
       break;
    end
end

clear vp;
```

Published with MATLAB® 7.13
