# Supplementary material for: Regulation of two motor patterns enables the gradual adjustment of locomotion strategy in Caenorhabditis elegans
Source: eLife. 2016 May 25;5:e14116. doi: 10.7554/eLife.14116 (PMC4880447; doi:10.7554/eLife.14116)
Supplement: Source code 1. — See ‘readme.txt’ for an overview. DOI: http://dx.doi.org/10.7554/eLife.14116.023 [file elife-14116-code1.zip › HumsSourceCode/ReadMe.rtf]

WormAngles
script RunAllSkeletons runs through all the codes to calculate angle time series of worm images 
(which are stored in the Parallel WormTracker format with additional modification to save binary images)
save data per experiment as WormGeomTracks.
Steps:
- FindAllWormSkeletonsFromTracks
  calculates the skeletons from binary worm images from tracks_als files.
- correctAngles
  smoothes skeleton, divides skeleton into segments of equal arc length and calculates angles in rad.
- HeadAssign;
  finds head coordinates of worm.
- skel_order;
  orders skeleton from head to tail and fixes left-right flips. 


EigenwormDecomposition
includes the following main scripts:

- GetEigenworm_fromCombinedTracks
  calculates eigenworms from input angle time series (from format: WormGeomTracks)

- EigenWormProjectiosScript_ontoFixedEWs
  projects angle time series onto input eigenworms and outputs time series of undulation mode and turning mode per track,
  saves them as EigenWormTracks; 
  calculates matrices of body, undulation and turning amplitudes;
  saves data from all experiments.

- EigenWormProjectiosScript_fixedEWs_forGradients_PerFile
  projects angle time series onto input eigenworms and outputs time series of undulation mode and turning mode per track,
  also saves body posture (= raw angle data)
  saves data per experiment as EigenWormTracks.
  Input for GradientAnalysis/ TurningMode/ BodyTurningxBearing

- EigenWormProjectiosScript_fxedEWs_PhaseSpeed
  calculates phase speed (= undulation frequency) by projecting angle time series onto input eigenworms.

Further this folder includes the decomposition sub-functions performing the eigenworm calculations;
and other used sub-functions.


Eigenmovie 
(main function: makeEigenMovie)
generates a movie showing an original video of a single worm from recorded assay 
and kymographs of body posture, undulation mode and turning mode indicating frames of the original worm video.
Further reconstructs and animates worm shapes based on full body posture, undulation mode and turning mode.


GradientAnalysis
- TurningMode/ BodyTurningxBearing:
  finds body turning events and bins them over bearing.
- Weathervaning-Speed-Reversals:
  folder contains several scripts binning reversal rate, speed or track curvature (=weathervaning) over bearing.


Used functions
contains mainly third party functions used within some of the codes.
